# An *IL28B* Genotype-Based Clinical Prediction Model for Treatment of Chronic Hepatitis C

**DOI:** 10.1371/journal.pone.0020904

**Published:** 2011-07-08

**Authors:** Thomas R. O'Brien, James E. Everhart, Timothy R. Morgan, Anna S. Lok, Raymond T. Chung, Yongwu Shao, Mitchell L. Shiffman, Myhanh Dotrang, John J. Sninsky, Herbert L. Bonkovsky, Ruth M. Pfeiffer, and the HALT-C Trial Group

**Affiliations:** 1 Division of Cancer Epidemiology and Genetics, National Cancer Institute, National Institutes of Health, Department of Health and Human Services, Bethesda, Maryland, United States of America; 2 Division of Digestive Diseases and Nutrition, National Institute of Diabetes and Digestive and Kidney Diseases, National Institutes of Health, Department of Health and Human Services, Bethesda, Maryland, United States of America; 3 Division of Gastroenterology, University of California Irvine, Irvine, California, United States of America; 4 Gastroenterology Service, VA Long Beach Healthcare System, Long Beach, California, United States of America; 5 Division of Gastroenterology, University of Michigan Medical Center, Ann Arbor, Michigan, United States of America; 6 Gastrointestinal Unit, Medical Services, Massachusetts General Hospital, Department of Medicine, Harvard Medical School, Boston, Massachusetts, United States of America; 7 Information Management Services, Silver Spring, Maryland, United States of America; 8 Liver Institute of Virginia, Bon Secours Health System, Newport News, Virginia, United States of America; 9 CSC, Rockville, Maryland, United States of America; 10 Celera Corporation, Alameda, California, United States of America; 11 Departments of Medicine and Molecular & Structural Biology and The Liver-Biliary-Pancreatic Center, University of Connecticut Health Center, Farmington, Connecticut, United States of America; 12 Carolinas Medical Center, Charlotte, North Carolina, United States of America; Karolinska Institutet, Sweden

## Abstract

**Background:**

Genetic variation in *IL28B* and other factors are associated with sustained virological response (SVR) after pegylated-interferon/ribavirin treatment for chronic hepatitis C (CHC). Using data from the HALT-C Trial, we developed a model to predict a patient's probability of SVR based on *IL28B* genotype and clinical variables.

**Methods:**

HALT-C enrolled patients with advanced CHC who had failed previous interferon-based treatment. Subjects were re-treated with pegylated-interferon/ribavirin during trial lead-in. We used step-wise logistic regression to calculate adjusted odds ratios (aOR) and create the predictive model. Leave-one-out cross-validation was used to predict a priori probabilities of SVR and determine area under the receiver operator characteristics curve (AUC).

**Results:**

Among 646 HCV genotype 1-infected European American patients, 14.2% achieved SVR. *IL28B* rs12979860-CC genotype was the strongest predictor of SVR (aOR, 7.56; p<.0001); the model also included HCV RNA (log10 IU/ml), AST∶ALT ratio, Ishak fibrosis score and prior ribavirin treatment. For this model AUC was 78.5%, compared to 73.0% for a model restricted to the four clinical predictors and 60.0% for a model restricted to *IL28B* genotype (p<0.001). Subjects with a predicted probability of SVR <10% had an observed SVR rate of 3.8%; subjects with a predicted probability >10% (43.3% of subjects) had an SVR rate of 27.9% and accounted for 84.8% of subjects actually achieving SVR. To verify that consideration of both *IL28B* genotype and clinical variables is required for treatment decisions, we calculated AUC values from published data for the IDEAL Study.

**Conclusion:**

A clinical prediction model based on *IL28B* genotype and clinical variables can yield useful individualized predictions of the probability of treatment success that could increase SVR rates and decrease the frequency of futile treatment among patients with CHC.

## Introduction

Chronic infection with hepatitis C virus (HCV) is an important cause of liver cancer and end-stage liver disease in the United States and worldwide [1], [2]. About 60–80% of persons who become infected with HCV fail to clear the virus spontaneously. Treatment with pegylated-interferon-alfa/ribavirin is associated with many adverse effects and results in a sustained virological response (SVR; i.e., undetectable HCV RNA six months post-treatment) in only 40–50% of interferon-naïve patients who are infected with HCV genotype 1 [3] (the most common viral genotype in the United States and many other developed countries) [4]. Recently, genome wide association studies (GWAS) found single nucleotide polymorphisms (SNPs) located upstream of *IL28B* (alternatively known as interferon-λ3) to be associated with SVR [5], [6], [7], [8]. *IL28B* variants are also associated with decreased frequency of spontaneous clearance of HCV [6], [9]. Interferon-λ induces the JAK/STAT pathway, which up-regulates genes with anti-viral effects against HCV [10], [11]. The newly identified SNPs likely mark a functional variant that affects response to interferon-α [12], [13].

A goal of genomic research is to yield information that leads to treatment decisions based on a patient's genetic makeup [14]. Personalized clinical decision-making for treatment of patients with chronic hepatitis C requires estimates of the probability that a patient will achieve SVR which consider not only *IL28B* genotype, but also other factors that are associated with treatment response [12]. Here we examine the association of *IL28B* genotype with response to treatment among participants in The Hepatitis C Antiviral Long-term Treatment against Cirrhosis (HALT-C) Trial, which enrolled patients with bridging fibrosis or cirrhosis who had not responded to previous interferon therapy [15]. We use these results to develop a model that predicts the individual probability of SVR for such patients based on genotype for the *IL28B* rs12979860 SNP and four commonly measured pre-treatment clinical variables.

## Methods

### Subjects

The design and primary results of the HALT-C Trial have been reported [15], [16]. Briefly, at enrollment, HALT-C patients had an Ishak fibrosis score ≥3 by local assessment of liver biopsy, had not previously responded to interferon treatment, had a Child-Turcotte-Pugh score <7 and had no evidence of hepatocellular carcinoma. Final assessment of fibrosis stage was performed by a panel of hepatopathologists [15], [16]. Patients with other liver diseases, human immunodeficiency virus infection, active illicit drug use or current alcohol abuse were excluded. During lead-in, patients received pegylated-interferon-α2a 180 mcg/week plus ribavirin 1.0–1.2 g/day. Subjects with undetectable HCV RNA at week 20 remained on combination treatment through week 48 and were followed until week 72. Subjects with undetectable HCV RNA at weeks 48 and 72 were considered to have an SVR.

Investigations of human genetics in the HALT-C Trial were conducted in those participants who provided (written) consent for genetic testing. Subjects who reported themselves to be ‘White,’ but not of Latino/Hispanic ethnicity, were termed ‘European American;’ those who reported themselves to be ‘White’ and of Latino/Hispanic ethnicity were termed ‘Hispanic;’ those self-reporting as Black were termed ‘African American.’ Subjects who did not report themselves to be in one of these three groups (n = 27) were excluded from this analysis. For external comparison, we genotyped *IL28B* SNPs in reference populations representative of European American or African American subjects (Text S1and Table S1).

The HALT-C Trial was approved by institutional review boards of the participating institutions: Human Subjects IRB, University of Massachusetts Medical Center, Worcester, MA; Human Subjects Protection Office, University of Connecticut Health Center, Farmington, CT; Biomedical IRB, Saint Louis University School of Medicine, St Louis, MO; Partners Human Research Committee, Boston, MA; Colorado Multiple Institutional Review Board, Aurora, CO; University of California - Irvine Institutional Review Board, Irvine, CA; IRB (Subcommittee on Human Studies), Long Beach VAMC Research Health Care Group, Long Beach, CA; Institutional Review Board, University of Texas Southwestern Medical Center, Dallas, TX; Institutional Review Board, University of Southern California, Los Angeles, CA; Institutional Review Board for Human Subject Research, University of Michigan Medical Center, Ann Arbor, MI; Office of Research Subjects Protection, Virginia Commonwealth University Health System, Richmond, VA; Institutional Review Board, National Institute of Diabetes and Digestive and Kidney Diseases, National Institutes of Health, Bethesda, MD; Institutional Review Board, New England Research Institutes, Watertown, MA.

### Laboratory

Serum HCV RNA and HCV genotype were determined as described previously (Text S1) [17]. The methods used to extract genomic DNA were described in an earlier publication [18]. Genotyping of *IL28B* SNPs was carried out by allele-specific real-time PCR [19] at a high throughput facility. For each allele-specific PCR reaction, 0.3 ng of DNA was amplified. Genotypes were automatically called by an in-house software program followed by manual curation without knowledge of phenotype. Primer sequences can be found in the Text S1.

### Statistical analyses

#### HALT-C Trial

Analyses of virological response were stratified by ethnicity and restricted to subjects who were infected with HCV genotype 1. To examine the effect of *IL28B* genotype over a range of possible virological responses, we divided patients into four mutually exclusive outcome groups based on serum HCV RNA levels during and after treatment: 1) null (<2 log_10_ IU/mL decrease at week 12); 2) partial early viral response (EVR) only (week 12: >2 log_10_ decrease; week 20: detectable); 3) relapse (week 20 and 48: undetectable; week 72: detectable) or breakthrough (week 20 undetectable; detectable sometime between week 20 and 48); 4) SVR (week 20, 48, and 72 undetectable). In these analyses, *IL28B* genotype frequencies were determined for each virological response group. Using null responders as the referent (i.e., baseline subject group) and homozygosity for the ancestral allele (rs12979860-T or rs8099917-T) as the baseline genotype, the genotype specific odds ratio (OR) and 95% confidence interval (CI) were determined.

Among European American patients who were infected with HCV genotype 1 (n = 646), we used a step-wise algorithm (selection criteria: entry, p = 0.10; exit, p = 0.05) to select variables for a logistic regression model that was used to calculate adjusted ORs and to estimate an individual's probability of achieving SVR or not. Candidate variables for this model were *IL28B* rs12979860 genotype and other factors that have been reported to be associated with SVR. All variables listed in Table S2 were entered into the step-wise model. Continuous variables were divided into categories with a minimum of 50 subjects per category (Table S2) and these variables were treated as ordinal predictors in the model, if appropriate. No variables were forced into the model, but, for the sake of comparison, we also created a model that was limited to the clinical variable and another that was limited to *IL28B* genotype. We used the likelihood ratio test to compare these models for fit of the data. To examine whether rs12979860 and rs8099917 were independently associated with virological response, we constructed models including both variants and compared them to single SNP models by the log likelihood test.

Among European American patients who were infected with HCV genotype 1 (n = 646), we used the leave-one-out cross-validation (LOOCV) method [20] to estimate the probability of SVR for each subject. A series of 646 logistic regression models was created with each model excluding a different subject from the dataset. An individual's probability of achieving SVR was obtained from the model to which they did not contribute, making these estimates unbiased *a priori* predictions of the probability of SVR.

We used the LOOCV predictions to estimate area under the ‘receiver operating characteristic’ curve (AUC), a popular measure of model discrimination [21]. To test for differences in AUC values between models, we computed a p-value based on a chi-square test (1 df) that used a bootstrap variance estimate computed by resampling the LOOCV predictions for subjects with SVR and non-responders, and then repeating the AUC computations for each bootstrap sample.

#### IDEAL Study

SVR rates according to the joint distribution of HCV RNA level (≤600,000, >600,000), METAVIR fibrosis score (F0–2, F3–4) and *IL28B* rs12979860 genotype (CC, CT, TT), have been published for 1,121 HCV-infected European American patients who enrolled in the IDEAL Study (found in Supplemental Table 4 of the paper by Thompson et al) [22]. The combination of these three risk factors can be viewed as a single predicator (X) with twelve (unordered) categories. We used Bayes's theorem to determine the distribution of X in IDEAL Study subjects who achieved SVR and in those who did not. We then randomly selected 10,000 values of X separately for IDEAL subjects who achieved SVR and from non-responders, and computed the logistic probability of SVR given X. We calculated AUC as the probability that the score for a randomly selected subject with SVR is greater than the score for a randomly selected non-responder, where score is the probability of SVR for a given X.

## Results

### Virological Response to Treatment in the HALT-C Trial

Demographic and clinical data at entry into HALT-C for all lead-in subjects who had an *IL28B* genotype result are shown in Table 1. Median age was 49 years, 72.4% were male, median pre-treatment HCV RNA was 6.5 log_10_ IU/ml; 37.6% had cirrhosis. Consistent with previous studies [5], [9], there was evidence for selection against the *IL28B* rs12979860-CC genotype among these patients, who had failed both to clear HCV spontaneously and to achieve SVR in response to prior therapy. *IL28B* rs12979860-CC frequency was 25.7% among 732 European American subjects in the Trial compared to 45.6% in the reference population (p<.0001) and 10.8% in 148 African American Trial subjects compared to 20.0% in the reference population (p = 0.01).

**Table 1:** Demographic and clinical characteristics of subjects in the HALT-C Trial study of *IL28B* genotype.

	All Lead-In Subjects (n = 992)		Prediction Model (n = 646)	
**Characteristic**				
**Age (median, IQR** a **)**	49	45–53	49	45–53
**Race**				
White (n, %)	732	73.8	**646**	100.0
Black (n, %)	148	14.9	0	
Hispanic (n, %)	85	8.6	0	
Other (n, %)	27	2.7	0	
**Male (n, %)**	718	72.4	487	75.4
**Ishak Fibrosis Score** b				
2 (n, %)	82	8.3	48	7.4
3–4 (n, %)	534	53.8	357	55.3
5–6 (n, %)	373	37.6	241	37.3
**HCV Genotype 1 (n, %)**	877	88.4	**646**	100.0
**HCV RNA level (log 10) (median, IQR** a **)**	6.5	6.1–6.8	6.5	6.2–6.8
**Prior treatment:**				
Interferon alone (n, %)	282	28.4	176	27.2
Interferon and ribavirin (n, %)	710	71.6	470	72.8

a IQR – Intra-quartile range (25th percentile–75th percentile).

b Final assessment of fibrosis stage was performed by a panel of hepatopathologists.

**Figure 1:**
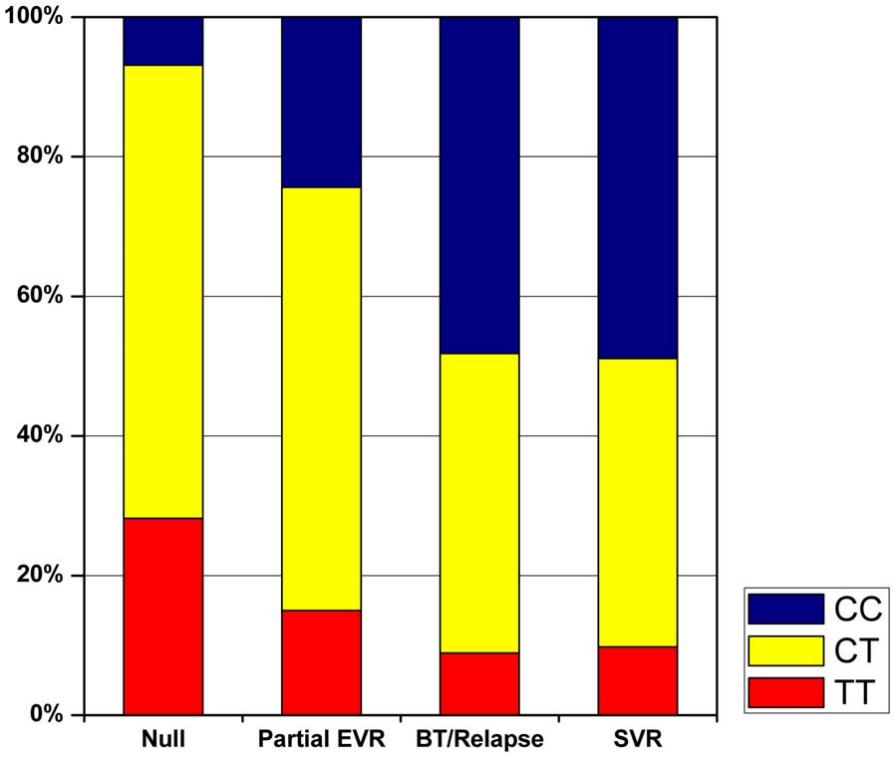
Distribution of *IL28B* rs12979860 genotypes, by response to treatment with pegylated-interferon-α2a plus ribavirin among European American subjects infected with HCV genotype 1, lead-in phase of the HALT-C Trial. EVR, early virological response; BT, breakthrough; SVR, sustained virological response.

Among European American patients who were infected with HCV genotype 1 (n = 646), the overall SVR rate was 14.2%. To examine the relationship between *IL28B* genotype and a range of virological responses, we performed an analysis among the 622 (96.3%) HCV genotype 1-infected European American patients who could be fully classified for virological response. *IL28B* rs12979860-CC frequency varied markedly by the degree of virological response (Figure 1; Table S3): null responders (referent), 6.9%; partial EVR, 24.4% (unadjusted OR,6.69; <.0001); breakthrough/relapse, 48.2%; SVR, 48.9%. The unadjusted OR observed for those with breakthrough/relapse and SVR were approximately 20 (p <.0001, each comparison), but the frequency of rs12979860-CC did not differ between these two groups even when other variables were considered in multivariate models (p = 0.56). Comparing subjects with undetectable HCV RNA at week 20 (breakthrough/relapse or SVR) to those with detectable virus (null or partial EVR) yielded adjusted ORs of 16.29 for rs12979860-CC (95% CI, 8.44–31.47; p<.0001) and 2.02 for rs12979860-CT (95% CI, 1.16–3.52; p = 0.01).

Among all European American subjects infected with HCV genotype 1, we compared *IL28B* genotype between those who achieved SVR and those who did not. Compared to rs12979860-TT, the OR for rs12979860-CC was 5.29 (95% CI, 2.46–11.38; p<.0001) and that for rs12979860-CT was 1.48 (95% CI, 0.69–3.16; p = 0.3). Fibrosis stage did not vary by rs12979860 genotype (mean and median = 4.0 for all three genotypes) in these subjects, but, consistent with prior reports [5], higher median pre-treatment HCV RNA levels were found in subjects with genotype rs12979860-CC (6.74 log_10_ IU/mL; p<.0001) and rs12979860–CT (6.50 log_10_ IU/mL; p = 0.04) compared to rs12979860-TT (6.41 log_10_ IU/mL). The logistic regression model included five variables: *IL28B* genotype (three categories), pre-treatment HCV RNA level (seven ordinal categories), AST/ALT ratio (five ordinal categories), Ishak fibrosis score (five ordinal categories) and prior treatment with ribavirin (yes/no). This model yielded an adjusted OR of 7.56 (95% CI, 3.20–17.87; p<.0001) for rs12979860-CC and an adjusted OR of 1.83 (95% CI, 0.82–4.11; p = 0.14) for rs12979860–CT.

The final model is described by this equation:

log odds = −2.8540−0.3532×fibrosis−0.4067×HCV RNA−0.4268×AST:ALT−0.6844×prior ribavirin+2.0226×*IL28B* rs12979860(CC)+0.6063×*IL28B* rs12979860(CT). Reference categories for co-variates are: HCV RNA = 6.50–6.74; AST∶ALT = 0.50–0.74, fibrosis = 3; prior ribavirin = yes; *IL28B* rs12979860(TT). On the basis of these log odds ratio parameter estimates, *IL28B* genotype was the strongest predictor of SVR in this study.

Of the 646 European American subjects infected with HCV genotype 1, 350 were maintained on at least 80% of full dose for both pegylated-interferon-α2a and ribavirin for the first 20 weeks of treatment. Among this ‘fully adherent’ subgroup, the association between *IL28B* genotype and SVR (rs12979860-CC: adjusted OR, 7.91; 95% CI, 2.80–22.35; p<.0001. rs12979860–CT: adjusted OR, 1.75; 95% CI, 0.67–4.57; p = 0.25) was similar to that found among HCV genotype 1-infected European American subjects as a whole.

Among 134 HCV genotype 1-infected African American patients, eight achieved SVR. For rs12979860-CC, the unadjusted OR was 12.69 (95% CI, 1.19–135.66; p = 0.04) and adjustment for covariates increased the association (adjusted OR, 48.02; 95% CI, 2.57–898.09; p = 0.01). The rs12979860-CC genotype was much more common in African American subjects with undetectable HCV RNA at week 20 (n = 21) than those with detectable virus (adjusted OR, 15.88; 95% CI, 2.90–86.96; p = 0.001). Hispanic subjects who were infected with HCV genotype 1 were too few (n = 67) for a meaningful analysis of virological response.

*IL28B* rs8099917 has been associated with response to treatment for chronic hepatitis C [5], [6], [7], [8] and this SNP is in strong linkage disequilibrium with rs12979860 [5]. Associations of rs8099917 genotype with virological response were similar to those for rs12979860, but models including both SNPs showed no independent effect of rs8099917 (data not presented).

### Prediction of Treatment Response in the HALT-C Trial

The subjects for the prediction model, European American patients who were infected with HCV genotype 1, were similar to HALT-C Trial lead-in subjects as a whole with regard to other demographic and clinical variables (Table 1). The distribution of *IL28B* rs12979860 genotypes among these 646 subjects was: CC, 24.0%; CT, 56.8%; TT, 19.2%.

**Figure 2:**
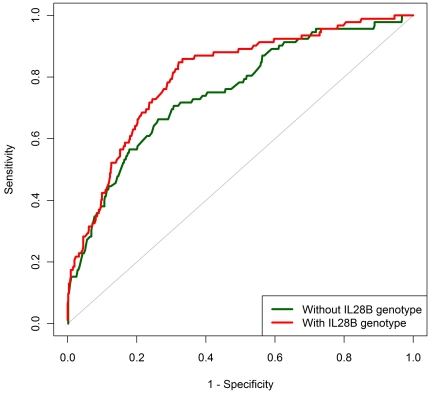
Area under the ‘receiver operating characteristic’ curve (AUC) for models predicting the probability of sustained virological response (SVR) - European American patients infected with HCV genotype 1, HALT-C Trial. Full model: *IL28B* rs12979860 genotype plus four clinical variables. AUC was calculated using predicted probabilities of SVR based on a series of leave-one-out cross-validation logistic regression models.

The logistic regression model based on *IL28B* genotype plus four clinical predictors of SVR (described above) fit the data better than models that included the four clinical predictors only or *IL28B* genotype only (p<0.001, both comparisons). For the full model (*IL28B* genotype plus clinical predictors), AUC was 78.5% compared to 73.0% for the model based on the clinical predictors without *IL28B* genotype (p<0.001; Figure 2). AUC was 60.0% for the model with *IL28B* genotype only.

**Figure 3:**
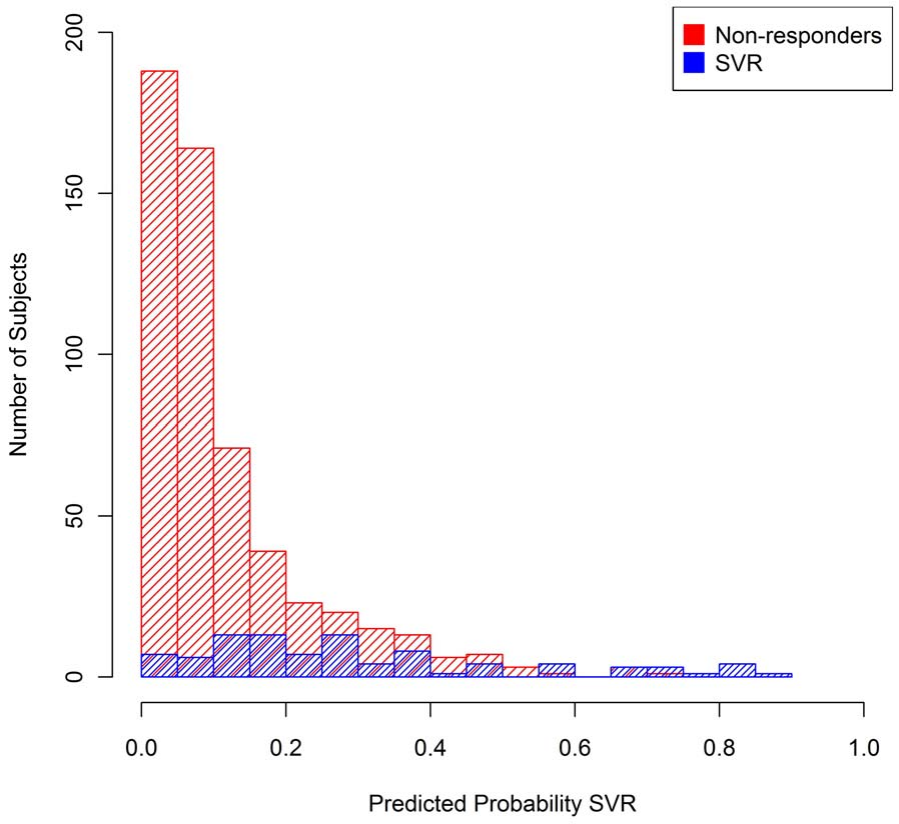
Distributions of *a priori* predicted probability of sustained virological response (SVR) for 554 non-responders and 92 subjects who achieved SVR - European American patients infected with HCV genotype 1, HALT-C Trial. Predicted probabilities are based on a series of leave-one-out cross-validation logistic regression models.

Based on the LOOCV models, 30.2% of these HALT-C subjects had an *a priori* predicted probability of achieving SVR <5%, 56.7% had a predicted probability <10%, 77.6% had a predicted probability <20% and 90.1% had a predicted probability <35% (Table 2). The distributions of *a priori* predicted probabilities of SVR differed markedly between the 554 non-responders and the 92 subjects who actually achieved SVR (Figure 3). For example, 36.5% of non-responders had a predicted probability ≥10%, compared to 84.8% of HALT-C subjects who achieved SVR. As a result of this relationship, the *IL28B* genotype-based model could predict which HALT-C subjects were more likely to achieve SVR. Table 2 shows SVR rates under a range of model-based treatment decision scenarios. For example, among the 280 HALT-C subjects with a predicted probability ≥10%, the observed SVR rate was 27.9%, compared to an SVR rate of 3.8% among the 366 patients with a predicted probability <10%. For HALT-C subjects, a strategy of treating those with a predicted probability ≥10% (and deferring treatment for the remaining subjects) would have yielded ∼85% of the total number of patients with SVR through treatment of 43% of the patients. Limiting treatment to those with a predicted probability ≥15%, would increase the SVR rate to 32.5% and decrease the number treated to 194 (30.0% of total subjects), while also decreasing the number who achieved SVR to 63 (68.5% of all subjects who actually achieved SVR).

**Table 2:** Number of subjects with various *a priori* predicted probabilities of SVR; projected SVR rates and numbers of patients achieving SVR if decision to treat was based on predicted probablity of SVR estimated from LOOCV models among EA patents infected with genotype 1, HALT-C Trial.

		Predicted Probability SVR											
		Any		5%		10%		15%		20%		35%	
	Treatment Decision Group	#	%	#	%	#	%	#	%	#	%	#	%
**≥Predicted Probability SVR**	**Treat**	646	100	451	69.8	280	43.3	194	30	145	22.4	64	9.9
**<Predicted Probability SVR**	**Defer**	0	0	195	30.2	366	56.7	452	70	501	77.6	582	90.1
**SVR Rate**	**Treat**		14.2		18.8		27.9		32.5		35.9		42.2
**SVR Rate**	**Defer**		N/A		3.6		3.8		6.4		8.0		11.2
**SVR Achieved**		92	100	85	92.4	78	84.8	63	68.5	52	56.5	27	29.3
**SVR Not Achieved**		0	0	7	7.6	14	15.2	29	31.5	40	43.5	65	70.7

**Figure 4:**
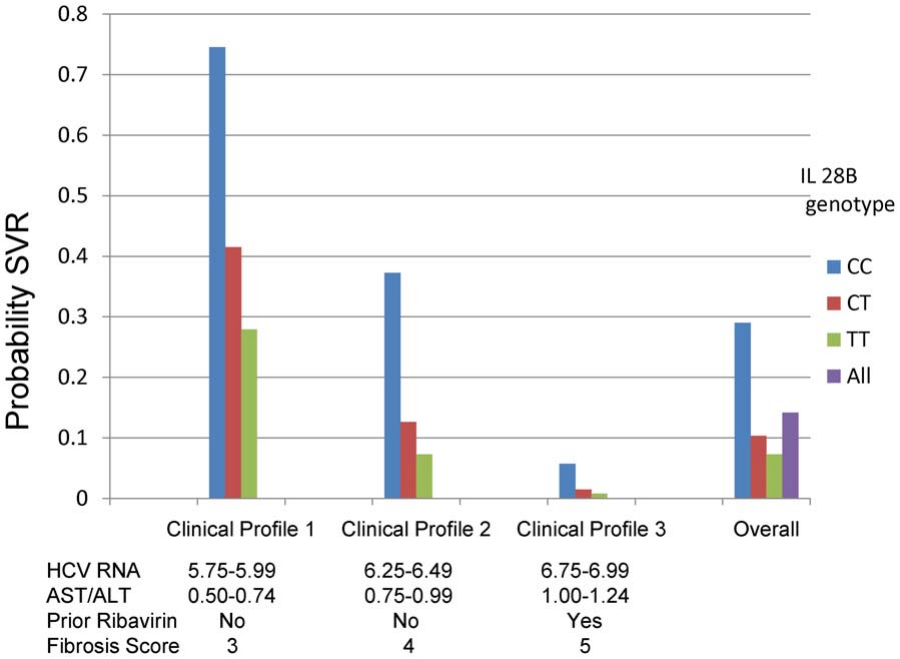
Observed and predicted probabilities of sustained virological response (SVR), by *IL28B* rs12979860 genotype, among European American patients infected with HCV genotype 1, HALT-C Trial. ‘Overall’ probabilities were directly observed. Probabilities for the selected clinical profiles were predicted from the model.

Figure 4 displays selected examples of SVR probabilities predicted by the model, as well as the observed SVR rates overall (14.2%) and by *IL28B* genotype (CC, 29.0%; CT, 10.3%; TT, 7.3%). Clinical profile 1consists of relatively favorable values for the four clinical predictors (HCV RNA, 5.75–5.99; AST/ALT, 0.50–0.74; no prior ribavirin; fibrosis score, 3). Among individuals with this clinical profile, the model predicted a probability of SVR as 74.5% for patients with the *IL28B*-CC genotype, 41.5% for *IL28B*-CT and 27.9% for *IL28B*-TT. Among individuals with the ‘intermediate’ clinical profile (Clinical profile 2: HCV RNA, 6.25–6.49; AST/ALT, 0.75–0.99; no prior ribavirin, fibrosis score, 4), the range in genotype-specific predicted probability of SVR was 30.0% (*IL28B*-CC, 37.3%; *IL28B*-CT, 12.6%; *IL28B*-TT, 7.3%). With a very unfavorable non-genetic profile (Clinical profile 3: HCV RNA, 6.75–6.99; AST/ALT, 1.00–1.24; prior ribavirin, fibrosis score, 5), the model predicted a very low SVR probability for all genotypes including *IL28B*-CC (5.7%).

### Prediction of Treatment Response in the IDEAL Study

To determine whether both *IL28B* genotype and clinical variables improve clinical prediction in a treatment naïve population, we used published data [22] among 1,121 HCV genotype 1-infected European American patients enrolled in the IDEAL study. The overall SVR rate in this population was 45.3%. AUC values were: *IL28B* genotype, HCV RNA and fibrosis, 73.7%; *IL28B* genotype, 67.8%; HCV RNA and fibrosis, 60.0% (all AUC values differ from each other, p<.01).

## Discussion

We demonstrated that *IL28B* genotype is a very strong predictor of SVR among patients with advanced chronic hepatitis C who failed previous therapy and then used these data to create a model to predict the probability of SVR based on *IL28B* genotype and selected clinical factors. The model must be validated in other populations before it can be implemented clinically, but our results provide ‘proof of concept’ that this approach has the potential to improve the care of patients with chronic hepatitis C. Patients with advanced chronic hepatitis C who have failed to respond to prior treatment for chronic hepatitis C have a very low rate of SVR overall, nonetheless the model identified patient profiles associated with a high probability of treatment response. As expected, most HALT-C subjects had a profile associated with a very low *a priori* probability of achieving SVR. Treatment decisions involve many considerations, but our work suggests that identifying patients with a low likelihood of success and advising them to await availability of more effective regimens could reduce the number of patients who would be exposed to a treatment with substantial adverse effects from which they will not derive a benefit, while having much less impact on the number who respond. For example, if treatment of HALT-C subjects had been limited to patients with a predicted probability ≥10%, 57% would have been spared treatment, the SVR rate would have been double and the number of subjects achieving SVR would have been 85% of that attained through treating the entire group.

Although *IL28B* genotype was the strongest predictor of SVR in this study, the prediction model was greatly improved by the inclusion of four parameters commonly available in clinical practice (pre-treatment HCV RNA level; AST/ALT ratio; fibrosis score; whether or not the patient received ribavirin during previous interferon-based treatment). In building the model, we divided continuous variables into multiple categories to avoid assumptions about the relationship between a variable and SVR based solely on the associations observed among HALT-C subjects. In addition, although the difference in SVR rate between rs12979860-CT and rs12979860-TT (adjusted OR, 1.83) did not reach statistical significance (p = 0.14), we retained all three *IL28B* genotype categories, which is consistent with the approach of some other groups [7]. The number of variables and categories in the model should not present a barrier to its clinical implementation, as these could be accommodated easily in a computer-based instrument.

Some variables previously associated with SVR were not selected into our logistic regression model. The effect of these variables may have been accounted for by variables in the model or our statistical power may have been inadequate to select these variables. Additional subjects might allow us to add variables to the model and improve its predictive ability.

Published data on joint SVR rates by *IL28B* genotype, HCV RNA level and fibrosis score (from the IDEAL study), allowed us to verify that AUC is increased among treatment naïve patients when both *IL28B* genotype and clinical variables are considered. However, individual patient data are required for our model and with additional data, our modeling approach could be extended. The HALT-C Trial was limited to subjects with advanced chronic hepatitis C who had failed to respond to previous interferon-based treatment. In developing the model, we further restricted subjects to those who were infected with HCV genotype 1 and of European ancestry because there were too few subjects in other ‘race’ or viral genotype groups for meaningful analysis. Given sufficient data, a prediction model for chronic hepatitis C treatment response could encompass the full range of HCV-infected patients, including those previously naïve for peg-interferon- alfa/ribavirin and those receiving regimens that include additional agents. Our modeling approach also could be expanded to include data on rapid virological response, but, unfortunately, those data are incomplete among HALT-C subjects. A ‘non-invasive’ prediction model that would not require measurement of fibrosis by liver biopsy might be desirable, but development and evaluation of such a model requires subjects encompassing the full range of fibrosis values.

Direct acting anti-viral agents that inhibit HCV replication, currently in late clinical development, promise to improve the SVR rate for patients who have failed to respond to treatment with interferon- alfa and ribavirin [23], as well as for patients who have not been treated for chronic hepatitis C previously [24], [25]. These compounds, which select drug-resistant HCV strains if used alone [26], will likely be combined with peginterferon alfa/ribavirin to reduce viral replication and mutational escape. Among patients who were treated with a regimen of peginterferon alfa/ribavirin plus the HCV-protease inhibitor telaprevir, 84% of those with the *IL28B* rs12979860-CC genotype achieved SVR compared to 32% among those with either *IL28B* rs12979860-CT or –TT [27]. Indirect evidence also suggests that *IL28B* genotype may be associated with response to a peginterferon alfa/ribavirin/telaprevir regimen. A recent trial among HCV-infected patients who had failed initial peginterferon alfa/ribavirin treatment found that retreatment with a regimen that included telaprevir was more effective among patients who had relapsed during previous treatment compared to previous nonresponders [23]. Given the strong association we observed between *IL28B* genotype and breakthrough/relapse, it is quite plausible that *IL28B* genotype is associated with response to a peginterferon alfa/ribavirin/telaprevir regimen. Therefore, an *IL28B* genotype-based model may identify patients who are at high risk for treatment failure (and selection of resistant HCV strains) when treated with this regimen.

Our work demonstrates that a model based on *IL28B* genotype and a few clinical variables can provide individualized predictions for the probability of achieving SVR after treatment with peg-interferon- alfa/ribavirin. If the *IL28B* genotype-based model is validated in a wide range of patients with chronic HCV infection, then development of a computer based algorithm for clinical decision making would seem warranted. Such a tool could improve patient outcomes among patients treated for chronic hepatitis C by increasing SVR rates and reducing the frequency of futile treatment, with its substantial costs and adverse effects.

## Supporting Information

Table S1 (DOC)

Table S2 (DOC)

Table S3 (DOC)

Text S1 (DOC)
