# Supplementary material for: An IL28B Genotype-Based Clinical Prediction Model for Treatment of Chronic Hepatitis C
Source: PLoS One. 2011 Jul 8;6(7):e20904. doi: 10.1371/journal.pone.0020904 (PMC3132753; doi:10.1371/journal.pone.0020904)
Supplement: Table S1 — (DOC) [file pone.0020904.s001.doc]

**An *IL28B* Genotype-Based Model for Prediction of**

**Response to Treatment of Chronic Hepatitis C**

Supplemental Table 1. Genotype frequencies for *IL28B* rs12979860 in European American and African American reference populations.

| **Ancestry** | **SNP** | **Genotype** | **Human Variation Panel** | | **Genomic Collaborative** | | **HAPMAP** | | **Total** | |
| --- | --- | --- | --- | --- | --- | --- | --- | --- | --- | --- |
| **European American** | **rs12979860** |  | No. | % | No. | % | No. | % | No. | % |
|  |  | CC | 86 | 46.7 | 142 | 44.5 | 28 | 47.4 | 256 | 45.6 |
|  |  | CT | 78 | 42.4 | 139 | 43.6 | 24 | 40.7 | 241 | 42.9 |
|  |  | TT | 20 | 10.9 | 38 | 11.9 | 7 | 11.9 | 65 | 11.6 |
|  |  | Total | 184 | 100.0 | 319 | 100.0 | 59 | 100.0 | 562 | 100.0 |
|  |  |  |  | |  | |  | |  | |
| **African American** | **rs12979860** |  |  |  |  |  |  |  |  |  |
|  |  | CC | 22 | 22.7 | 71 | 19.4 |  |  | 93 | 20.0 |
|  |  | CT | 43 | 44.3 | 162 | 44.1 |  |  | 205 | 44.2 |
|  |  | TT | 32 | 33.0 | 134 | 36.5 |  |  | 166 | 35.8 |
|  |  | Total | 97 | 100.0 | 367 | 100.0 |  |  | 464 | 100.0 |
