# Supplementary material for: An IL28B Genotype-Based Clinical Prediction Model for Treatment of Chronic Hepatitis C
Source: PLoS One. 2011 Jul 8;6(7):e20904. doi: 10.1371/journal.pone.0020904 (PMC3132753; doi:10.1371/journal.pone.0020904)
Supplement: Table S2 — (DOC) [file pone.0020904.s002.doc]

Supplemental Table 2. Frequency and rate of SVR by demographic characteristics, clinical predictors of virological response and IL28B genotype, among European American participants in the lead-in phase of the HALT-C Trial who were infected with HCV genotype 1.

|  | **Subjects** | | **SVR** |
| --- | --- | --- | --- |
|  | **#** | **%** | **Rate** |
| **Overall** | 646 | 100.00% | 14.20% |
|  |  |  |  |
| **Gender** |  |  |  |
| Male | 487 | 75.4% | 15.4% |
| Female | 159 | 24.6% | 10.7% |
|  |  |  |  |
| **Age** |  |  |  |
| <44 | 119 | 18.4% | 18.5% |
| 45-49 | 236 | 36.5% | 16.5% |
| 50-54 | 169 | 26.1% | 12.4% |
| 55-59 | 65 | 10.1% | 10.8% |
| 60+ | 57 | 8.8% | 5.3% |
|  |  |  |  |
| **BMI** |  |  |  |
| 17.5-24.9 | 115 | 17.8% | 14.8% |
| 25.0-29.9 | 267 | 41.3% | 14.2% |
| 30.0-58.4 | 264 | 40.9% | 14.0% |
|  |  |  |  |
| **Ishak Fibrosis Score** |  |  |  |
| 2 | 48 | 7.4% | 31.3% |
| 3 | 244 | 37.8% | 16.0% |
| 4 | 113 | 17.5% | 15.0% |
| 5 | 130 | 20.1% | 9.2% |
| 6 | 111 | 17.2% | 8.1% |
|  |  |  |  |
| **Steatosis** |  |  |  |
| 0 | 129 | 20.0% | 18.6% |
| 1 | 263 | 40.7% | 14.4% |
| 2 | 197 | 30.5% | 12.2% |
| 3-4 | 57 | 8.8% | 10.5% |

| **HCV RNA level** |  |  |  |
| --- | --- | --- | --- |
| 3.03-5.74 | 63 | 9.8% | 34.9% |
| 5.75-5.99 | 52 | 8.0% | 21.2% |
| 6.00-6.24 | 79 | 12.2% | 13.9% |
| 6.25-6.49 | 111 | 17.2% | 13.5% |
| 6.50-6.74 | 143 | 22.1% | 7.0% |
| 6.75-6.99 | 123 | 19.0% | 13.8% |
| 7.00-7.63 | 75 | 11.6% | 8.0% |
|  |  |  |  |
| **ALT** |  |  |  |
| <50 | 99 | 15.3% | 9.1% |
| 50-99 | 265 | 41.0% | 12.8% |
| 100-149 | 155 | 24.0% | 12.3% |
| 150-199 | 66 | 10.2% | 27.3% |
| 200+ | 61 | 9.4% | 19.7% |
|  |  |  |  |
| **AST_ALT** |  |  |  |
| <.50 | 43 | 6.7% | 32.6% |
| 0.50-0.74 | 254 | 39.3% | 19.7% |
| 0.75-0.99 | 214 | 33.1% | 9.8% |
| 1.00-1.24 | 80 | 12.4% | 3.8% |
| 1.25+ | 55 | 8.5% | 7.3% |
|  |  |  |  |
| **Glucose intolerance** |  |  |  |
| Normal (<100 mg/dl) | 363 | 56.2% | 16.5% |
| Impaired (100-125 mg/dl) | 146 | 22.6% | 13.7% |
| Provisional Diabetes (126+ mg/dl) or | 137 | 21.2% | 8.8% |
| Diagnosed Diabetes |  |  |  |

|  |  |  |  |
| --- | --- | --- | --- |
| **Prior treatment:** |  |  |  |
| Interferon alone | 176 | 27.2% | 23.3% |
| Interferon and ribavirin | 470 | 72.8% | 10.9% |
|  |  |  |  |
| **rs12979860** |  |  |  |
| CC | 155 | 24.0% | 29.0% |
| CT | 367 | 56.8% | 10.4% |
| TT | 124 | 19.2% | 7.3% |

Continuous variables were divided into categories (minimum of 50 subjects per category) based on arithmetic cut points
