# Supplementary material for: An IL28B Genotype-Based Clinical Prediction Model for Treatment of Chronic Hepatitis C
Source: PLoS One. 2011 Jul 8;6(7):e20904. doi: 10.1371/journal.pone.0020904 (PMC3132753; doi:10.1371/journal.pone.0020904)
Supplement: Table S3 — (DOC) [file pone.0020904.s003.doc]

**An *IL28B* Genotype-Based Model for Prediction of**

**Response to Treatment of Chronic Hepatitis C**

Supplemental Table 3. Genotype frequencies for *IL28B* rs12979860 and unadjusted odds ratios, by response to treatment with pegylated-interferon-α2a/ribavirin during the lead-in phase of the HALT-C Trial among European American infected with HCV genotype 1. P_MH_ is overall p-value.

|  |  | **Null** | | | **Partial EVR** | | | **Relapse** | | | **SVR** | | |
| --- | --- | --- | --- | --- | --- | --- | --- | --- | --- | --- | --- | --- | --- |
| **SNP** | **Genotype** | **#** | **%** |  | **#** | **%** | **OR** | **#** | **%** | **OR** | **#** | **%** | **OR** |
| **rs12979860** | CC | 20 | 6.9 |  | 31 | 24.4 | 6.69 | 54 | 48.2 | 22.14 | 45 | 48.9 | 20.50 |
|  | CT | 189 | 64.9 |  | 77 | 60.6 | 1.76 | 48 | 42.9 | 2.08 | 38 | 41.3 | 1.83 |
|  | TT | 82 | 28.2 |  | 19 | 15.0 |  | 10 | 8.9 |  | 9 | 9.8 |  |
|  | Total | 291 | 100.0 |  | 127 | 100.0 |  | 112 | 100.0 |  | 92 | 100.0 |  |
|  | P_MH_ |  |  |  |  | <.0001 |  |  | <.0001 |  |  | <.0001 |  |
