# Supplementary material for: An IL28B Genotype-Based Clinical Prediction Model for Treatment of Chronic Hepatitis C
Source: PLoS One. 2011 Jul 8;6(7):e20904. doi: 10.1371/journal.pone.0020904 (PMC3132753; doi:10.1371/journal.pone.0020904)
Supplement: Text S1 — (DOC) [file pone.0020904.s004.doc]

**An *IL28B* Genotype-Based Model for Prediction of**

**Response to Treatment of Chronic Hepatitis C**

**Supplemental Text**

**Methods**

*Subjects*

The reference population for European American subjects was comprised of: 319 samples from throughout the United States obtained by Genomics Collaborative, INC. (GCI); 186 North American samples obtained from the National Institute of General Medical Science (NIGMS) Human Variation Panels (HVP); and a reference panel of 59 individuals from Utah with ancestry from northern and southern Europe originally (International HapMap Project). The reference population for African American subjects included: 367 African Americans from throughout the United States obtained by GCI and 98 African Americans obtained from NIGMS HVP. GCI obtained informed written consent from all participants and local institutional review board approval at all recruitment sites.

*Laboratory*

Serum HCV RNA was measured by Roche COBAS™ Amplicor HCV Monitor, v.2.0 (analytic sensitivity, 600 IU/ml; Roche Molecular Systems, Branchburg, NJ) and, if undetectable, by Roche COBAS Amplicor HCV Test, v.2.0 (analytic sensitivity, 100 IU/ml). HCV genotype was determined with the INNO-LiPA HCV II kit (Siemens Medical Solutions Diagnostics, Tarrytown, NY).

rs12979860 Genotyping

Allele specific primer sequence:

AS1: GCAATTCAACCCTGGTTCG

AS2: GCAATTCAACCCTGGTTCA

Common primer sequence:

GCTTATCGCATACGGCTAGGC

rs8099917 Genotyping

Allele specific primer sequence:

AS1: GTTTTCCTTTCTGTGAGCAATG

AS2: TGTTTTCCTTTCTGTGAGCAATT

Common primer sequence:

GATGTGGGAGAATGCAAATGAGAGAT
